# Real-Time On-Device Continual Learning Based on a Combined Nearest Class Mean and Replay Method for Smartphone Gesture Recognition

**DOI:** 10.3390/s25020427

**Published:** 2025-01-13

**Authors:** Heon-Sung Park, Min-Kyung Sung, Dae-Won Kim, Jaesung Lee

**Affiliations:** 1 School of Computer Science and Engineering, Chung-Dang University, Heukseok-dong, Dongjak-gu, Seoul 06974, Republic of Korea; hopo55@cau.ac.kr; 2 Department of Artifcial Intelligence, Chung-Ang University, Heukseok-dong, Dongjak-gu, Seoul 06974, Republic of Korea; clary9752@gmail.com

**Keywords:** gesture recognition, on-device AI, continual learning

## Abstract

Sensor-based gesture recognition on mobile devices is critical to human–computer interaction, enabling intuitive user input for various applications. However, current approaches often rely on server-based retraining whenever new gestures are introduced, incurring substantial energy consumption and latency due to frequent data transmission. To address these limitations, we present the first on-device continual learning framework for gesture recognition. Leveraging the Nearest Class Mean (NCM) classifier coupled with a replay-based update strategy, our method enables continuous adaptation to new gestures under limited computing and memory resources. By employing replay buffer management, we efficiently store and revisit previously learned instances, mitigating catastrophic forgetting and ensuring stable performance as new gestures are added. Experimental results on a Samsung Galaxy S10 device demonstrate that our method achieves over 99% accuracy while operating entirely on-device, offering a compelling synergy between computational efficiency, robust continual learning, and high recognition accuracy. This work demonstrates the potential of on-device continual learning frameworks that integrate NCM classifiers with replay-based techniques, thereby advancing the field of resource-constrained, adaptive gesture recognition.

## 1. Introduction

Gesture recognition is key to advancing human–computer interaction by translating physical movements into intuitive commands or delivering information [1]. The increasing prevalence of smartphones, smartwatches, and other wearable devices equipped with sensors has led to the broad applicability of gesture recognition across a range of domains, including robotics, virtual reality, and smart home environments [2]. Deep learning models have become the standard approach for automatically extracting features and recognizing complex patterns in multichannel sensor data [3]. Traditionally, training and inference have been conducted on remote servers, requiring that raw sensor data be periodically transmitted from the device to the server, where updated models are retrained and sent back to the device [4]. As user-defined gestures evolve and new ones are frequently introduced, such as replacing a circular gesture for increasing TV volume with a more convenient swipe gesture, frequent server-based retraining becomes necessary, leading to substantial power consumption, communication overhead, and latency [5].

Existing mainstream methods for gesture recognition rely heavily on computationally intensive server-based architectures, presenting several limitations. First, they require frequent data transmission between devices and servers, resulting in high energy consumption and potential data privacy concerns. Second, these methods often depend on complex neural networks with large memory footprints, making them impractical for resource-constrained devices like smartphones. Finally, most approaches suffer from catastrophic forgetting when learning new gestures incrementally, necessitating frequent retraining that is both computationally expensive and inefficient.

Continual learning (CL) techniques address the challenge of incrementally learning new classes and instances without retraining from scratch, demonstrating remarkable advancements in computer vision tasks [6]. By extending these methods to on-device gesture recognition, we can continuously adapt deep learning models to new gestures from streaming sensor data. However, current approaches fail to offer a practical solution for mobile devices, as they struggle to balance computational efficiency, memory limitations, and accuracy in the face of incremental updates.

To overcome these limitations, we propose the first on-device continual learning framework for gesture recognition. Key innovations include the use of a lightweight Nearest Class Mean (NCM) classifier combined with replay-based updates, which together minimize computational overhead and memory usage. Additionally, we introduce a novel buffer management strategy that prioritizes representative samples, ensuring efficient use of limited memory resources. These improvements enable our method to achieve high accuracy and stability while addressing the main drawbacks of existing approaches, such as high computational complexity and catastrophic forgetting.

In this work, we build upon the principles introduced by on-device continual learning methodologies, including the use of NCM classifiers and replay-based updates. Inspired by these strategies, we propose a novel on-device continual learning framework specifically tailored for gesture recognition. The proposed framework excludes fully connected classification heads in favor of an NCM classifier, simplifying computations and reducing energy consumption. To counteract the tendency of the NCM classifier to underperform due to its simplicity, we integrate replay-based updates that strategically reintroduce previously seen samples, preventing catastrophic forgetting and enhancing classification accuracy. Furthermore, replay buffer management techniques are employed to optimize the utilization of the device limited memory resources, maintaining a balanced subset of historical data for continual adaptation.

Our main contributions are as follows:

- We present the first on-device continual learning framework specifically for resource-constrained gesture recognition, utilizing an NCM classifier and replay-based updates to learn new gestures in real time without server interaction.
- We introduce replay buffer management strategies to preserve and reuse samples from previously learned gestures efficiently, thereby maintaining robust accuracy and mitigating forgetting.
- We implemented and evaluated our method on a commercial smartphone (Samsung Galaxy S10), which demonstrated stable on-device operation with high recognition accuracy (approximately 99%), thereby confirming the viability and effectiveness of our approach.
- This paper extends these aspects of our previous work in ICCE 2024.

## 2. Related Works

Gesture recognition plays a pivotal role in advancing human–computer interaction, functioning as a natural interface that bridges the gap between users and devices. Traditional methods in this domain often rely on visual cues, such as camera-based tracking or computer vision models, to interpret complex hand movements and body gestures [1,2]. While these approaches have benefitted from advancements in deep learning, they can still be hindered by variable lighting conditions, occlusions, and privacy concerns, especially in practical, real-world settings.

In response to these challenges, researchers have increasingly focused on sensor-based gesture recognition. By utilizing inertial measurement units (IMUs), including accelerometers and gyroscopes, these methods can robustly capture the subtle dynamics of movement, offering improved resilience to environmental changes. This shift toward sensor-based solutions has led to a burgeoning body of work exploring sophisticated modeling techniques. Early efforts involved classical machine learning approaches, such as Dynamic Time Warping (DTW) or Hidden Markov Models (HMMs), to capture temporal dynamics from time-series data [7,8]. More recently, deep learning architectures, especially Convolutional Neural Networks (CNNs) and Recurrent Neural Networks (RNNs), have emerged as the backbone of sensor-based gesture recognition, automatically extracting high-level features and learning complex temporal dependencies [3,9,10,11,12,13].

Beyond accuracy improvements, interpretability and robustness have become significant research focal points in the sensor-based domain. Some studies introduced geometric and morphological considerations to better understand how gestures vary across individuals and contexts [14]. Others explored sensor fusion strategies, combining accelerometers, gyroscopes, and even radar or photosensor data to improve robustness against noise, environmental interference, and device orientation [15,16]. Meanwhile, transfer learning and domain adaptation techniques have been investigated to reduce training burdens and handle discrepancies between training and test conditions [17,18].

Recent works, such as X-DER [6], have highlighted the importance of episodic memory updates to counter catastrophic forgetting effectively. X-DER introduces techniques to incorporate information about unseen classes into memory, enabling a gradual adaptation of memory representations as new tasks emerge. Additionally, strategies like future preparation, which involve initializing future classification heads to accommodate unseen data distributions, have proven effective in improving generalization and mitigating forgetting in class-incremental continual learning scenarios. These innovations illustrate a shift toward memory-efficient and adaptive continual learning frameworks, offering robust solutions in resource-constrained environments.

As gesture recognition technology matures, attention has turned toward efficiency, scalability, and adaptability. One major hurdle is the need for models that can dynamically update and refine their knowledge as new gestures emerge, a critical capability in scenarios where user preferences or environmental contexts change frequently. Continual learning (CL) methods present a promising avenue, allowing models to incrementally learn new tasks or classes without catastrophic forgetting of previously acquired knowledge. Key approaches in CL, such as iCaRL [19], have introduced replay-based strategies that integrate memory buffers to store representative samples from past classes. Subsequent improvements have employed techniques like supervised contrastive replay [20], gradient episodic memory (GEM) [21,22], and regularization-based methods [23,24], often benchmarked on image classification tasks.

In parallel, there has been a growing body of work focusing on adapting continual learning methods to resource-constrained environments and non-vision modalities. Approaches designed for low-compute or on-device scenarios aim to overcome the computational and memory limits of mobile devices, employing quantization, pruning, and other compression strategies [25,26]. For instance, latent replay methods [27], smartphone-oriented incremental learning [28], and scalable frameworks like Deep SLDA [29] have explored ways to incrementally update models on-device while preserving prior knowledge. Though most of these advances have been demonstrated in image-based applications, a few studies have begun exploring incremental learning for non-vision tasks such as human activity recognition and surface electromyography (sEMG) gesture recognition [30,31,32]. These pioneering efforts have highlighted the potential benefits of applying continual learning to sensor-based tasks but have not fully addressed the unique challenges of real-time, on-device adaptation for gesture recognition.

Additionally, research on vision-based incremental gesture recognition, such as class-incremental learning on egocentric videos or adapting to environmental variability [5,33,34], offers valuable insights. However, the modality gap between images and sensor data means that these methods cannot be directly transferred without considerable modifications. Existing studies that address incremental learning in sensor-based scenarios often focus on large-scale server-side training or rely on model retraining when new classes appear.

In summary, while the literature on gesture recognition and continual learning is extensive, few works have directly targeted the challenge of on-device continual adaptation for sensor-based gestures. Current methods typically emphasize either efficient on-device inference or server-side incremental retraining, leaving a gap for frameworks that can continuously adapt to new gestures locally without catastrophic forgetting or frequent offloading. The present work addresses this gap by combining NCM-based classification with replay buffer management, enabling a lightweight, robust, and memory-efficient approach that runs entirely on-device. By doing so, we contribute to bridging the divide between gesture recognition evolving practical demands and the advanced capabilities offered by continual learning frameworks.

## 3. Proposed Method

**Figure 1:**
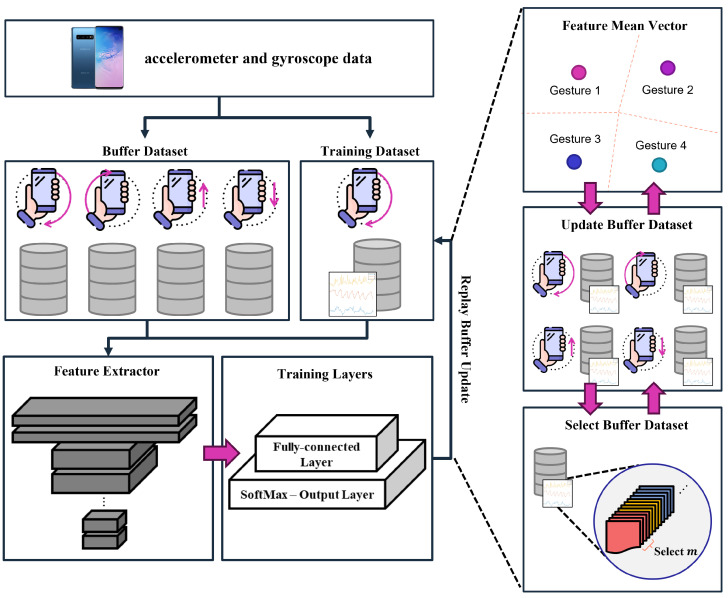
The overall procedure of the on-device continual learning framework for gesture recognition. This framework enables continual learning on mobile devices through two main elements: network learning and replay buffer management technology.

In this section, we present our on-device continual learning framework tailored for sensor-based gesture recognition in resource-constrained environments. The primary objective of the proposed method is to enable continuous adaptation to new gestures introduced over time, while ensuring minimal forgetting of previously learned classes and maintaining efficient use of computational and memory resources. To achieve this, our framework integrates three key components: (1) a preprocessing pipeline that standardizes varying-length time series sensor data for consistent feature extraction, (2) a continual learning strategy employing a Nearest Class Mean (NCM) classifier with replay-based updates to mitigate catastrophic forgetting, and (3) a replay buffer management technique that balances memory utilization and recognition accuracy. Figure 1 illustrates an overview of the proposed framework.

Developing a continual learning framework for on-device gesture recognition entails addressing several critical challenges. First, raw sensor signals representing user gestures (e.g., accelerometer or gyroscope readings) are often irregular in length and noisy, complicating feature extraction. Second, updating the model in real time as new gestures are introduced must be performed efficiently, as fully retraining complex models on limited hardware may lead to excessive latency and energy consumption. Third, replay-based methods, while effective in maintaining previously learned knowledge, must be carefully managed to fit within the tight memory budgets of mobile devices. Our approach effectively tackles each of these issues.

In the process of designing this framework, the following three issues should be addressed:

- Extracting gesture features is challenging because users have varying speeds and trajectories when performing gestures, resulting in data collections of differing lengths.
- Training new gestures through complex deep learning models can be time-consuming and challenging to perform in real time due to the limited computational resources of mobile devices.
- The memory capacity of devices such as the Samsung Galaxy S10 is limited; it must be possible to simultaneously learn data from the replay buffer and new gesture data.

### 3.1. Time Series Transformation

A fundamental step in our framework is converting the variable-length time series sensor data into a fixed-size representation. With this step, achieving consistent feature extraction would be easier due to unpredictable gesture durations and user-dependent variations in gesture speed and trajectory. We utilize a cubic interpolation method to resample the raw data into a uniform length. Specifically, given a sequence of sensor readings, we fit cubic polynomials between data points to estimate intermediate values. This results in a regularized time series that retains the key features of the gesture while normalizing its length. By performing this preprocessing, we simplify subsequent feature extraction and ensure that the network can focus on meaningful patterns rather than handling variable input shapes. This approach, while computationally light, captures both subtle variations and overarching trends in the gesture signals, ultimately supporting more robust and efficient model updates. This transformation is a way to minimize information loss in time series data, as shown in Equation (1) as follows:

(1) $$f\left(x\right)=mx^{3}+nx^{2}+jx+k$$

where m,n,j represents the coefficient for each order, *k* is a constant term, and *x* is the data point.

Cubic interpolation uses four data points to interpolate a value at a point between the second and the third data points. For each segment between data points, solve for the coefficients m,n,j, and *k* such that the cubic polynomial passes through the known data points. Use the cubic polynomial equations to find the new interpolated points at the desired equally spaced intervals. Using cubic polynomials to interpolate between points results in smooth curves connecting each point. However, if the continuity of the derivatives is not maintained, there may be sharp changes in the curve at these points, which may not accurately represent the underlying data. We let the function pass through two points and ensure that the first and second derivatives remain the same with the condition as follows:

(2) $$f\left(x_{0}\right)=y_{0}\;\mathrm{and}\;f\left(x_{1}\right)=y_{1}$$

(3) $$f^{\prime }\left(x_{0}\right)=f^{\prime }\left(x_{1}\right)\;\mathrm{and}\;f^{\prime \prime }\left(x_{0}\right)=f^{\prime \prime }\left(x_{1}\right)$$

The time series data processed according to Equation (1) preserve the main characteristics of the original data and exhibit robustness to noise. In addition, it is relatively simple while maintaining high precision compared with computationally complex interpolation methods, making it helpful in capturing the data change rate more accurately. The fixed-size time series data converted in this manner are used as input to a CNN. A CNN has the ability to automatically extract features of various sizes and characteristics from the data, thereby facilitating more effective feature extraction from such converted data. This approach allows for a more efficient and precise analysis of time series data.

### 3.2. Feature Extraction and Classifier Design

At the core of our framework lies a feature extractor pretrained on a source dataset. This extractor, implemented as a series of convolutional layers, automatically distills complex sensor signals into compact feature embeddings. Rather than continually adjusting the weights of the feature extractor, which would require significant computational overhead and risk of catastrophic forgetting, we employ a fixed feature extractor. By freezing these layers, we eliminate the need for computationally expensive backpropagation through the entire network during incremental updates. Instead, we focus on adjusting the parameters of a lightweight NCM classifier, which assigns class labels based on proximity to class-mean feature vectors.

The NCM classifier offers a streamlined classification strategy that does not rely on retraining a complex, fully connected layer. Instead, we store and update class mean vectors as new gestures are introduced. When a new sample arrives, we compute its embedding through the fixed feature extractor and update the corresponding class mean. During inference, classification decisions are made by selecting the class whose mean vector is closest to the input embedding in the feature space. This design drastically reduces training time and computational cost, allowing the system to adapt to new gestures in nearly real time on resource-limited hardware.

### 3.3. Replay-Based Continual Learning Strategy

While the NCM classifier provides a simple and efficient mechanism for incremental updates, it alone cannot prevent catastrophic forgetting of previously learned gestures. We designed a fast and efficient gesture recognition learning method optimized for mobile environments with memory constraints by integrating a replay-based continual learning strategy.

**Figure 2:**
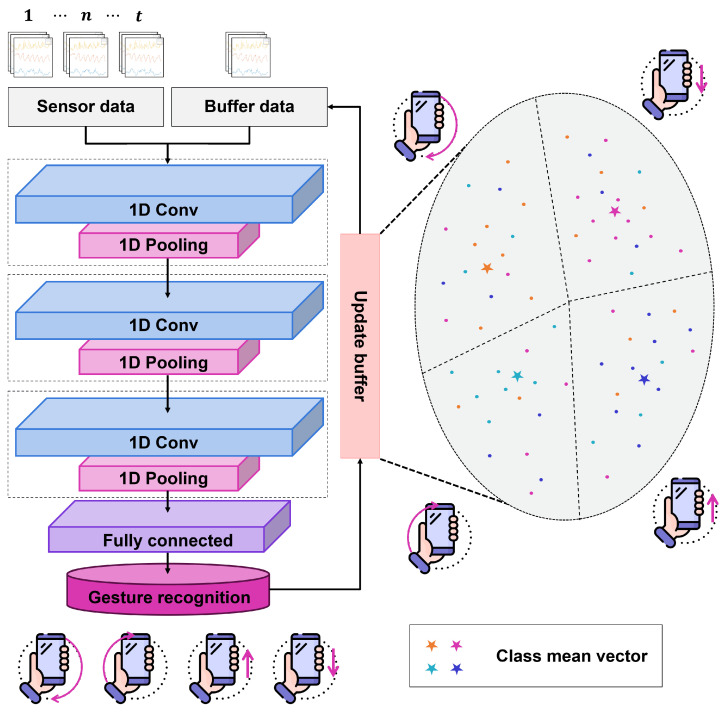
Structure of the gesture recognition network using sensor data, with a replay buffer for continual learning of new gestures through 1D convolutional layers and a fully connected layer.

When new gesture data are introduced, the primary features of the gesture are quickly extracted using a fixed feature extractor. These extracted features serve as input to the subsequent processing stage, where additional learning is confined to the fully connected layer, which is the final component of the deep learning model. The newly collected gesture data are further processed by extracting features from the source domain $D_{s}$ and aligning them with the specific new training task $T_{n}$. Through this process, efficient learning of new gestures is achieved. Figure 2 shows our gesture recognition network, which processes sensor data through 1D convolutional layers and a fully connected layer while employing a replay buffer for continual learning and retention of previously acquired knowledge.

To ensure stability during the introduction of a new gesture class, we combine its samples with a small subset of previously encountered samples stored in a replay buffer. This replay mechanism helps the classifier retain familiarity with older classes, preventing the mean embeddings of these classes from drifting excessively, which could otherwise degrade classification accuracy. A key challenge in continually learning new gestures is the tendency to forget information about previously learned gestures. To address this issue, we implemented a replay-based continual learning method. This approach effectively mitigates information loss by storing representative samples of previously learned data and integrating them with new data during training. Furthermore, for devices with limited memory capacity, in this case the Samsung Galaxy S10, we developed an efficient strategy to simultaneously process data from the replay buffer and the new gesture data, ensuring optimal memory utilization while maintaining high recognition accuracy.

**Figure 3:**
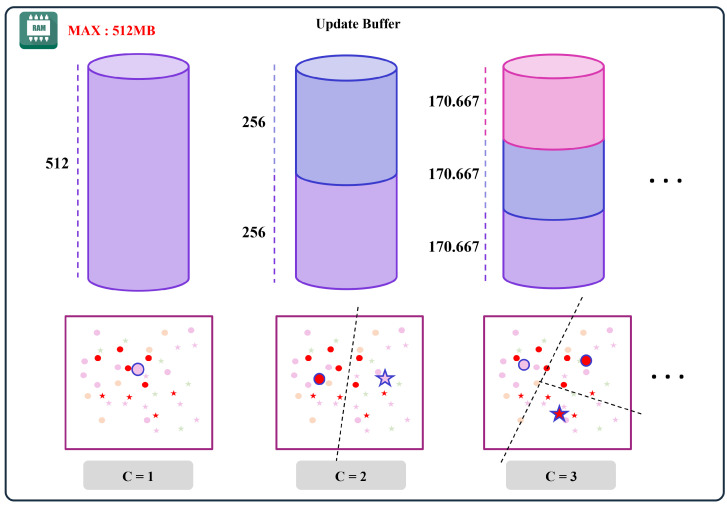
Replay buffer management technology to minimize mobile device memory usage. This technique stores some data similar to the feature mean vector of the new gesture while not exceeding memory usage.

Figure 3 depicts the replay buffer management strategy, which stores samples nearest each class mean vector to ensure efficient memory usage on resource-constrained devices. Specifically, when a new gesture class arrives, we invoke an update routine that: (1) extracts features for the new gesture samples using the fixed feature extractor, (2) adjusts the NCM classifier means to reflect the new class, and (3) retrains the classifier parameters using both the new class data and replay samples from previously learned classes. By carefully balancing old and new samples, the framework preserves earlier knowledge while accommodating the newly introduced gesture. Detailed explanations are provided in Algorithms 1–4.

Our method calls the update routine Algorithm 1 whenever new gesture data become available. In this update process, the classifier is trained using the feature vector of the training sample $D_{t}=\left\{X_{t},Y_{t}\right\}$ of task *t*, which is sequentially processed through a fixed feature extractor, where, $X_{t}$ represents the input data (i.e., raw sensor signals), $Y_{t}$ is the corresponding label (the specific gesture class), *t* is the number of tasks or classes learned so far, and the fixed feature extractor $\varphi :X\to R^{d}$ transforms raw inputs into concise, meaningful feature embeddings. The classifier then utilizes a trainable fully connected layer, whose parameters are denoted by θ. This setup allows the system to seamlessly integrate new gestures without retraining the entire network or relying on a remote server. During the learning process, an appropriate buffer size *K* is set, considering the memory constraints of the mobile device. When a new gesture is introduced, the replay buffer is dynamically resized: stored samples of previously learned gestures are reduced by *m* units to free up space, and samples of the new gesture are added. This ensures that the buffer size remains uniform across all classes, maintaining knowledge of old gestures while allowing the device to adapt efficiently to new gestures within its memory limitations.
**Algorithm 1** Training Process of the On-Device Continual Learning**Input:**  $D_{t}$   ▹ training data of current task**Input:** *K*          ▹ replay buffer size    **Require:** θ  ▹ current model parameters    **Require: **$P=(P_{1},\cdots ,P_{t-1})$   ▹ current buffer dataset    $D_{t}=\left\{X_{t},Y_{t}\right\}$    θ← Update Model Parameters$\left(D_{t},P,\theta \right)$    m=K/t      ▹ buffer size per class    P← CommentReduce Buffer Dataset(P,m,t)    $P_{t}\leftarrow$ Construct Buffer Dataset$\left(X_{t},m,\theta \right)$    $P\leftarrow (P_{1},\cdots ,P_{t})$      ▹ new buffer dataset

**Figure 4:**
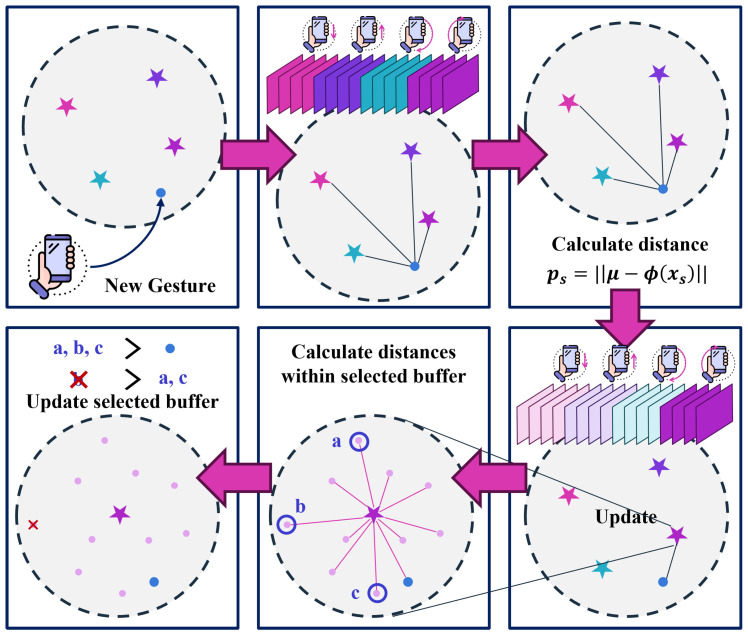
Replay buffer management process for continual learning. When a new gesture is introduced, its representative features are calculated and stored in the buffer. The system removes the buffer data farthest from the representative features to maintain fixed memory usage while preserving knowledge of previously learned gestures.

Figure 4 illustrates how new representative features are added and less representative ones removed, maintaining fixed memory usage while preserving knowledge of prior gestures. Algorithm 2 describes the process of updating θ using feature vectors extracted from the new gesture data. This procedure combines the current training samples with the stored buffer data to form an extended training dataset. If no previously learned gestures exist (i.e., the buffer is empty), the method relies solely on the current training dataset. Subsequently, the model uses this extended dataset as input and infers the output, which is then evaluated through a loss function to measure its performance. By minimizing this loss, θ is iteratively updated to enhance the model predictive accuracy. This approach ensures that newly introduced gestures are learned in the context of previously acquired knowledge. It enables the model to refine its understanding of past gestures while adapting to new ones, without forgetting prior classes. This design allows on-device training to remain both incremental and efficient, preventing excessive memory use.
**Algorithm 2** Update Model Parameters**Input: **$D_{t}$
▹ training dataset    **Require: **θ▹ current model parameters    **Require: **$P=(P_{1},\cdots ,P_{t-1})$  ▹ current buffer dataset    $D_{t}=\left\{X_{t},Y_{t}\right\}$
    **if** t>1$\;D\leftarrow \left\{\left(x,y\right):x\in X_{y}\right\}\cup \underset{y=1,\cdots ,t}{⋃}\left\{\left(x,y\right):x\in P_{y}\right\}$    **else**$\;D\leftarrow \{\left(x,y\right):x\in X_{y}\}$    **end**    $\ell \left(\varphi_{\theta }\right)=\sum_{i=1}^{t}\ell \left(\varphi \left(D_{t,x};\theta \right),D_{t,y}\right)$
    $g\leftarrow \nabla \ell (\varphi_{\theta })$
    θ←θ−αg**Output:** 
θ


### 3.4. Replay Buffer Management

Memory constraints pose a significant challenge in on-device learning, requiring efficient management of the replay buffer. Storing a large number of samples for each class becomes infeasible as the number of learned classes increases. To address this limitation, we propose a replay buffer management technique that selects only the most representative samples per class, minimizing memory usage without compromising performance.

Our approach maintains and continually updates a class mean vector derived from the embeddings of each class. To determine which samples to retain, we calculate the embedding distance of each candidate sample from the class mean vector and select those most similar to the mean. These representative samples effectively capture the core characteristics of the class, ensuring that the replay buffer retains high-quality exemplars that preserve the class identity over time.

When a new class is introduced, we dynamically adjust the buffer size per class to maintain balanced memory allocation. Outdated or redundant samples that lack meaningful representational value are discarded. This strategy enables our method to scale gracefully, accommodating an increasing number of gestures without overwhelming the limited memory resources of on-device systems.

Algorithm 3 provides a detailed procedure for efficiently managing the buffer dataset under strict memory constraints. Let *t* be the number of tasks learned so far and *K* be the maximum total number of samples that can be stored. Whenever new gesture data arrive, the method recalculates the per-class sample limit $m=\frac{K}{t}$. This mechanism automatically redistributes the available memory as more classes emerge, preventing any single class from monopolizing the buffer.
**Algorithm 3** Reduce Buffer Dataset**Input:** *m*
     ▹ buffer size per class**Input:** *t*      ▹ number of tasks**Require:** $P=(P_{1},\cdots ,P_{t-1})$     ▹ current buffer dataset   **for** n=1,⋯,t−1
**do**      $P_{n}\leftarrow P_{n}=\left(p_{1},\cdots ,p_{m}\right)$     ▹ reduce buffer dataset   **end for****Output:** buffer dataset *P*

To capture the essence of each class, the algorithm retains only the *m* samples that are most similar to that class mean vector. Typically, similarity is measured by the Euclidean distance between the feature embedding of a sample and the class mean vector. By selecting these representative samples, the system preserves the key characteristics of each gesture class while adhering to the current memory limit. In practical terms, this means that, as new classes accumulate, older classes retain only their most informative samples, thus mitigating catastrophic forgetting while keeping storage usage under control.

Algorithm 4 details how representative samples are selected to update the buffer dataset. Given a fixed feature extractor, we first compute the mean vector μ for the current class by averaging the feature embeddings of its samples. Next, the entire dataset is sorted according to the similarity (often measured by Euclidean distance) of each sample embedding to μ. Only the top *m* samples those closest to μ are kept, and the rest are discarded. This ensures that the buffer dataset *P* is refreshed with the most informative data for newly introduced gestures.
**Algorithm 4** Construct Buffer Dataset**Input:** $X=\{x_{1},\cdots ,x_{n}\}$           ▹ training data of current task**Input:** 
*m*    **Require:** $\varphi :X\to R^{d}$            ▹ current feature function    $\mu \leftarrow \frac{1}{n}\sum_{x\in X}\varphi \left(x\right)$            ▹ current class mean    **for** s=1,⋯,n **do**$\;p_{s}\leftarrow ∥\mu -\varphi \left(x_{s}\right)∥$    **end for**    P←argsort(P)    $P\leftarrow (p_{1},\cdots ,p_{m})$**Output:** buffer dataset *P*

This selection step enables the system to preserve high-quality exemplars that best represent each gesture class. By keeping only the samples most similar to the class mean, the method prevents the buffer from becoming cluttered with redundant or outlier data. Such a strategy is particularly valuable in on-device scenarios, where memory is limited; focusing on a condensed but informative set of samples helps maintain recognition performance without overburdening the system resources.

This replay buffer management technique maximizes the retention of previously learned information by prioritizing representative samples instead of random selection. Consequently, it enables mobile devices to continuously and efficiently learn new gestures while maintaining high accuracy and allowing immediate updates.

## 4. Experimental Results

In this section, we verify the performance of on-device continual learning for gesture recognition. First, we describe the experiment setup, including the pretraining dataset, custom user dataset, feature extraction network, scenario, and implementation details. The following section presents performance comparison results for two scenarios that involve continuous gesture recognition.

### 4.1. Experimental Setup

This section outlines the datasets, data preprocessing, and experimental design used to validate our proposed framework for gesture recognition.

#### 4.1.1. Datasets

6DMG Dataset: The 6DMG dataset contains data from 20 distinct gesture classes collected using a Wii Remote device equipped with accelerometer and gyroscope sensors [10]. Data were recorded at a sampling rate of 60 Hz from a diverse group of participants, with each gesture start and end points defined by users. Each gesture class includes approximately 280 samples, culminating in a total of 5615 samples. To simulate incremental learning, we begin with a single gesture class and sequentially add one new class at a time until all 20 classes are included. At each incremental step, 80% of the data is used for training while 20% is reserved for validation, enabling evaluation of the model ability to recognize both old and newly added classes.

PUG Dataset: The PUG dataset was collected using a Samsung Galaxy S10 smartphone to evaluate the proposed method under realistic on-device conditions. It comprises four gesture classes performed by a single user at a sampling rate of 100 Hz. Although the smartphone orientation was kept consistent during data collection, the execution style and speed of each gesture were left unconstrained to emulate natural usage. Each gesture class includes 600 samples, with 300 collected using the left hand and 300 using the right hand, resulting in a total of 2400 samples across all classes. Similar to the 6DMG dataset, incremental learning is simulated by starting with one gesture class and adding one class at a time until all four classes are included. In each incremental step, 80% of the samples is allocated for training, and 20% is used for validation to monitor how well the model accommodates new classes without forgetting previously learned ones.

#### 4.1.2. Data Preprocessing

- Segment Size and Interpolation:We use a fixed segment size of 250 for both datasets. If a gesture sequence is shorter than 250, cubic interpolation is applied to fill missing values; if it is longer, only the first 250 timesteps are used.
- Normalization: We apply BatchNormalization layers within the neural network to normalize input channels (accelerometer, gyroscope) and stabilize training.
- Data Augmentation: No augmentation is applied, to preserve original gesture characteristics and reduce potential bias.

#### 4.1.3. Experimental Design

- Continual Learning Setup: Each experiment starts with a single gesture class, incrementally adding one new class until all classes are learned.
- Class Imbalance: Class imbalance is not a concern in these datasets, as each gesture class contains an approximately equal number of samples. No additional resampling methods are employed.
- Evaluation Metrics: After each incremental step, we evaluate classification accuracy on both previously learned and newly added classes to quantify the model capacity for knowledge retention and adaptability.

This experimental design mirrors real-world scenarios where new gestures are gradually introduced over time, highlighting the scalability and efficiency of the proposed on-device framework under limited memory and computational constraints. By avoiding retraining from scratch, the method demonstrates a practical approach to continual learning for gesture recognition.

We utilized a feature extractor composed of convolutional layers from a deep learning model pretrained on our method to facilitate fast gesture recognition on mobile devices with limited resources. We compared using three feature extraction networks as follows:

- CNN(+Ours): A network was proposed that recognizes gestures by automatically extracting the local features of gestures that perform short actions [11]. It automatically extracts local gesture features and uses them to update our method buffer.
- 2-Stream CNN(+Ours): A network was proposed that obtains separate weight values for the accelerometer and gyroscope features through different convolution layers [12]. This network utilizes different feature information to update the buffer of our method effectively.
- CNN-GRU(+Ours): A network was proposed that considered both local and global features of gestures [13]. This network can effectively update the buffer of our method by considering local and global features.

We set up two continual learning scenarios as follows:

- Class Incremental Scenario: We set up a scenario where new gestures are continuously added over time. An experiment was conducted to evaluate whether the network could sequentially learn the four gestures in the PUG dataset and retain information about these learned gestures.
- Instance Incremental Scenario: We set up a scenario where new samples of existing gestures are added over time instead of introducing new gestures. We initially trained the model on four gestures from the PUG dataset collected with the left hand, followed by training on new samples collected with the right hand to evaluate performance differences.

All networks were implemented using TensorFlow. Subsequently, the network was converted using TensorFlow Lite to facilitate operation on the Samsung Galaxy S10. We developed an application for gesture recognition that can run on a Samsung Galaxy S10 equipped with the Android OS. All learning and testing processes are conducted using a mobile device CPU.

To ensure that our method could operate under realistic mobile device constraints, we allocated a maximum memory budget of 512 MB for our on-device continual learning experiments. Notably, despite this allocation, the actual memory consumption remained substantially lower during runtime. This efficient memory usage indicates that the proposed framework can be deployed on devices with even stricter resource limitations, such as tiny sensors or wearable devices.

### 4.2. Comparison Results

In the class-incremental scenario, we investigated how the system learned new gestures sequentially and retained knowledge of previously learned gestures. As shown in Table 1, the baseline CNN-based approaches suffer pronounced drops in F1-score when new gestures are introduced. A simple CNN without continual learning mechanisms achieves only 8.6–29.99% accuracy across the four gestures, which illustrates severe forgetting of earlier classes. By contrast, our proposed replay-enhanced strategy dramatically improves performance. In the CNN variant augmented with our approach, F1-scores increase to a range of 98.33–100%. The 2-Stream CNN and CNN-GRU architectures exhibit a similar trend. Their baseline scores range between 2.5% and 85.84%, yet they soar to well above 96% when our method is integrated.

X-DER, which is recognized as a strong baseline for incremental learning, demonstrates an average F1-score of approximately 98.62% after learning all four gestures, as shown in Table 2. Even so, our methods surpass X-DER in final performance. The 2-Stream CNN configuration enhanced with our replay mechanism reaches 99.17%, which is about 0.55 percentage points higher than X-DER. This configuration also shows minimal performance degradation at each incremental step. Likewise, our single-stream CNN and CNN-GRU setups maintain higher accuracy than X-DER. The integrated replay buffer usage and richer feature extraction help mitigate catastrophic forgetting and accelerate adaptation to newly introduced gestures.

Training and inference times indicate that our methods are suitable for on-device deployment, as shown in Table 2. The single-stream CNN variant typically takes between 1 and 2 s for training when introducing each new gesture, while the 2-Stream CNN and CNN-GRU approaches require about 2 to 5 s. Inference remains well below one second for all models. Although X-DER also operates efficiently, our experiments suggest that the proposed replay-based framework achieves a more favorable trade-off between recognition accuracy and real-time performance demands.

When new samples of previously known gestures are introduced, such as right-hand variations after training on left-hand data, our approaches again exhibit superior stability compared to X-DER. Table 3 shows that X-DER performance declines by an average of 1.54 percentage points in this instance-incremental scenario. Meanwhile, the 2-Stream CNN configuration only experiences a 0.42 percentage point drop, and the single-stream CNN sees a 0.52 drop. These small reductions highlight the ability of our replay-based mechanism to adapt to novel conditions without sacrificing prior knowledge.

Table 4 provides further insights by illustrating how increasing the replay buffer size enhances accuracy for all methods. Our approaches often match or exceed X-DER performance even at smaller buffer sizes of 100 to 200 samples. This result underscores the effectiveness of well-structured replay strategies and continual regularization. Efficient memory usage is especially crucial for mobile or embedded systems where on-device storage is limited.

From a theoretical standpoint, the replay method helps mitigate catastrophic forgetting by routinely reintroducing previously learned samples, ensuring that the model continues to reinforce knowledge of earlier classes. In addition, adopting a Nearest Class Mean (NCM) approach offers advantages under low-memory conditions. This approach employs an online averaging mechanism to update class prototypes while maintaining time complexity at O(1). There is no need to store or revisit all historical data, making the method especially memory-efficient for on-device incremental learning.

**Figure 5:**
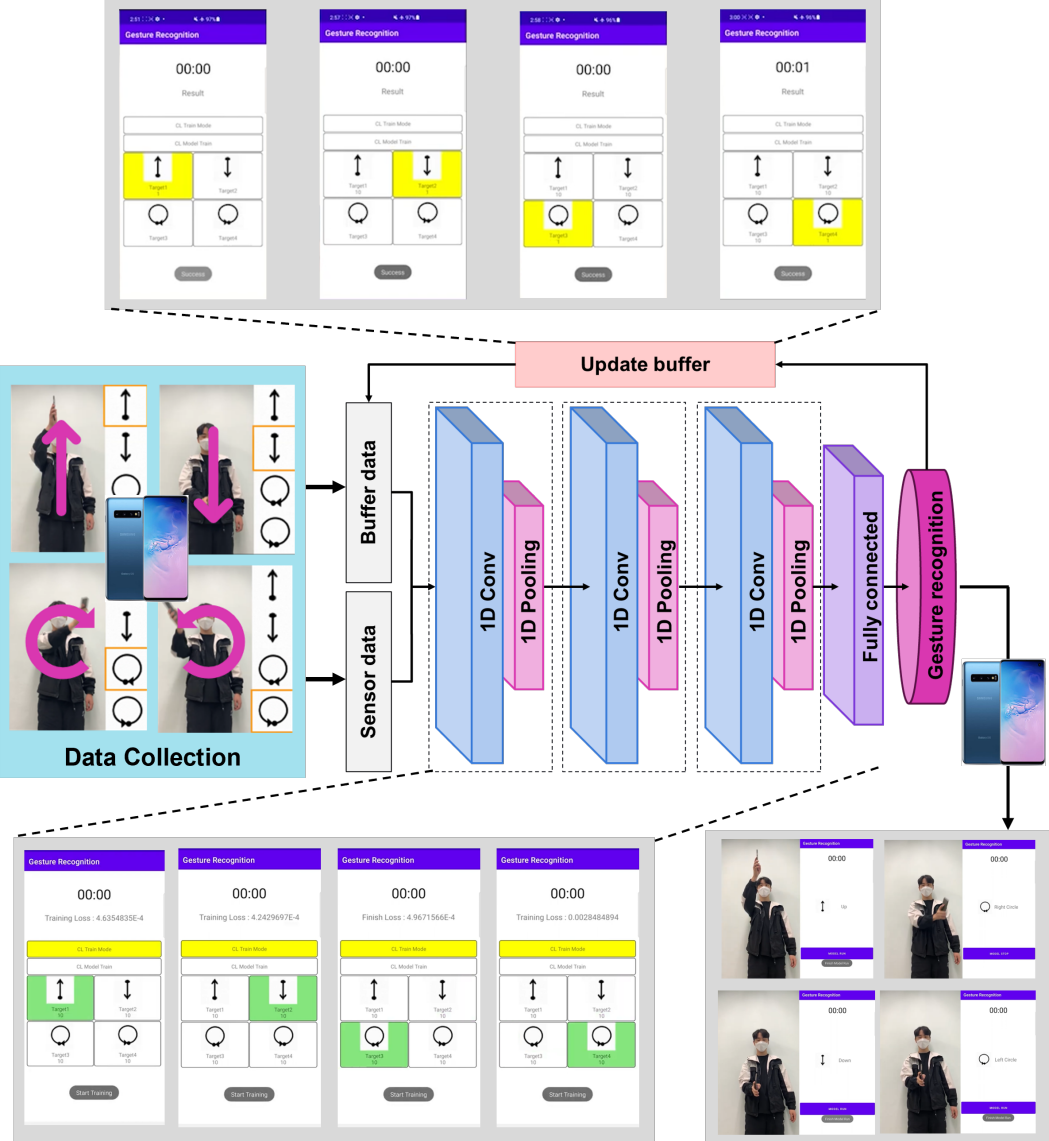
Results of our method performing gesture recognition on a Samsung Galaxy S10 smartphone. The figure demonstrates the system successful real-time operation and incremental learning of gestures, highlighting its practical functionality through a dedicated mobile application.

Overall, these results confirm that our replay-enhanced models offer robust performance in both class-incremental and instance-incremental learning, achieve rapid on-device training and inference, and make efficient use of available memory resources. By improving upon the X-DER baseline in all of these aspects, our approach demonstrates its suitability for real-world, resource-constrained scenarios. Figure 5 demonstrates on-device incremental learning on a Galaxy S10, enabling continuous adaptation without server retraining.

## 5. Discussion

The experimental results demonstrate the efficacy and feasibility of the proposed on-device continual learning framework for gesture recognition under resource-constrained mobile environments. By employing a Nearest Class Mean (NCM) classifier and a replay-based continual learning strategy, our method successfully mitigates the issue of catastrophic forgetting and achieves high accuracy, consistently surpassing 99%. This success is achieved without resorting to frequent data transfers to remote servers, demonstrating how incremental adaptation to new gesture classes can be maintained locally on mobile devices.

A key insight from our findings is that the framework preserves performance across both class-incremental and instance-incremental scenarios. Among the tested feature extraction architectures, the 2-Stream CNN with our method maintained the highest average accuracy and demonstrated robust retention of previously learned classes. This indicates that leveraging multiple sensor modalities (for instance, accelerometer and gyroscope) via separate convolutional streams produces more comprehensive feature representations. Such representations help counteract the potential loss of accuracy when introducing new gestures or encountering variations in user execution styles.

Central to the framework efficiency is the replay buffer management, which carefully selects representative samples based on their proximity to class mean vectors rather than randomly. This approach ensures that stored samples truly encapsulate previously learned distributions, allowing the device to manage its limited memory more effectively. Notably, while we initially allocated a maximum memory size of 512 MB for these experiments, the actual memory utilization proved to be substantially lower. This efficiency gain is significant and demonstrates that our method can be adapted to extremely memory-constrained environments, making it suitable for tiny devices or wearable sensors with far stricter memory budgets.

The robustness of our method in such constrained conditions underscores its potential in resource-limited applications such as Internet of Things (IoT) sensor nodes, miniature wearables, and other embedded systems. Future research could explore the scalability of the replay buffer, optimal selection strategies, and compression techniques to further reduce storage requirements. Although our method successfully handles new classes, subtle performance degradation can occur if users drastically change their gesture execution style. This points to future avenues of research, such as incorporating meta-learning or active learning approaches that enable rapid model calibration and personalized on-device fine-tuning.

Another important direction for future work involves a detailed analysis of energy consumption during continuous on-device training. Since many mobile and IoT devices rely on limited battery capacities, optimizing power usage is crucial to sustaining continuous learning. Profiling and reducing CPU, GPU, and memory usage during replay and model updates will be essential for wide-scale, long-term deployments. In addition, validating our approach across diverse populations remains an open challenge. Extensive user studies will help confirm whether the framework generalizes well under different skill levels, hand sizes, and execution styles.

Finally, there is also a need to investigate the optimal hyperparameters related to both the replay buffer and the feature extractor network. Although we have demonstrated that even moderate buffer sizes yield high accuracy, systematically exploring how memory allocations, buffer update frequencies, and feature extractor configurations trade off against each other is a rich area for further work. Identifying configurations that balance accuracy, memory usage, inference latency, and training time could lead to more refined on-device learning strategies.

## 6. Conclusions

In this paper, we introduced the On-Device Continual Gesture Recognition System, the first on-device continual learning framework designed for the resource constraints of mobile devices. By applying a preprocessing step to convert variable-length time series data into a uniform format, we enabled efficient and robust feature extraction from raw sensor inputs. Leveraging the Nearest Class Mean (NCM) classifier alongside replay-based continual learning strategies, our method successfully mitigates catastrophic forgetting, ensuring that previously learned gestures are retained while adapting to new gestures in real time. A key advantage of our approach lies in its effective memory management scheme, particularly relevant for devices with limited resources. Despite allocating a maximum memory budget of 512 MB, our system consumed considerably less memory in practice, suggesting that our method can be deployed on even smaller devices with stricter resource constraints, such as tiny or wearable sensor nodes. This efficiency does not compromise accuracy; on the contrary, our experiments on the Samsung Galaxy S10 demonstrate that our method can achieve over 99% recognition accuracy while performing both training and inference entirely on-device.

In the future, several research directions remain to be explored. For instance, as the number of gestures grows, developing more effective replay buffer management strategies will be crucial for maintaining balanced performance without exceeding memory limits. In addition, exploring adaptive techniques such as meta-learning or active learning to handle variations in user behavior and evolving usage scenarios can further enhance personalization and robustness. Investigating compression techniques or hierarchical memory structures may also improve scalability, ensuring that our method continues to operate efficiently as devices and applications evolve. In conclusion, our work demonstrates that on-device continual learning for gesture recognition is not only feasible but also highly performant and resource efficient. This paves the way for advanced, user-adaptive, and available gesture recognition solutions that run on various resource-constrained devices, from smartphones to tiny devices.

## Figures and Tables

**Table 1:** Class-incremental gesture recognition: comparison of average F1-score results across different models (Gesture 1 = Up, Gesture 2 = Down, Gesture 3 = Circle Right, Gesture 4 = Circle Left).

Model	Gesture 1	Gesture 2	Gesture 3	Gesture 4
CNN [11]	29.99	9.17	9.16	8.6
CNN [11] (+Ours)	98.33	99.17	98.33	100
2-Stream CNN [12]	31.66	3.33	2.5	24.16
2-Stream CNN [12] (+Ours)	100	99.17	97.5	100
CNN-GRU [13]	8.34	1.67	8.3	85.84
CNN-GRU [13] (+Ours)	99.17	98.33	96.67	99.17

**Table 2:** Class-incremental scenario: detailed F1-score performance, training time, and inference time per incremental task (Gesture 1 = Up, Gesture 2 = Down, Gesture 3 = Circle Right, Gesture 4 = Circle Left).

Model	Task	Gesture 1	Gesture 2	Gesture 3	Gesture 4	Average	Training Time (ms)	Inference Time (ms)
X-DER [6]	1st	100.00	-	-	-	100.00	3318	606
	2nd	98.33	99.83	-	-	99.08	6250	654
	3rd	99.33	98.67	98.67	-	98.89	7302	630
	4th	98.33	99.67	97.33	99.17	98.62	10034	692
CNN [11] (+Ours)	1st	100.00	-	-	-	100.00	1123	183
	2nd	98.33	100.00	-	-	99.17	1357	106
	3rd	99.17	99.17	100.00	-	99.45	1531	116
	4th	98.33	99.17	98.33	100.00	98.96	2103	139
2-Stream CNN [12] (+Ours)	1st	100.00	-	-	-	100.00	1237	264
	2nd	100.00	99.17	-	-	99.59	1687	220
	3rd	100.00	96.67	96.67	-	97.78	3015	275
	4th	100.00	99.17	97.50	100.00	99.17	4125	288
CNN-GRU [13] (+Ours)	1st	100.00	-	-	-	100.00	1659	303
	2nd	97.50	100.00	-	-	98.75	3125	327
	3rd	99.17	95.83	98.33	-	97.78	3651	315
	4th	99.17	98.33	96.67	99.17	98.34	5017	346

**Table 3:** Instance-incremental scenario: F1-score results when new right-hand instances are added after training on left-hand gestures (Gesture 1 = Up, Gesture 2 = Down, Gesture 3 = Circle Right, Gesture 4 = Circle Left).

Model	Target	Left Hand	Right Hand	Total	Performance Difference
X-DER [6]	Gesture 1	99.50	97.67	98.59	−1.83
	Gesture 2	98.67	98.33	98.50	−0.34
	Gesture 3	98.33	97.67	98.00	−0.66
	Gesture 4	99.67	98.33	99.00	−1.34
	Average	99.04	97.50	98.27	−1.54
CNN [11] (+Ours)	Gesture 1	99.17	97.50	98.34	−0.83
	Gesture 2	98.33	98.33	98.33	-
	Gesture 3	99.17	98.33	98.75	−0.42
	Gesture 4	100	98.33	99.17	−0.84
	Average	99.17	98.12	98.65	−0.52
2-Stream CNN [12] (+Ours)	Gesture 1	99.17	98.33	98.75	−0.42
	Gesture 2	99.17	98.33	98.75	−0.42
	Gesture 3	97.50	96.67	97.09	−0.41
	Gesture 4	100	99.17	99.59	−0.41
	Average	98.96	98.13	98.55	−0.42
CNN-GRU [13] (+Ours)	Gesture 1	100	98.33	99.17	−0.84
	Gesture 2	99.17	98.33	98.75	−0.42
	Gesture 3	99.17	96.67	97.92	−1.25
	Gesture 4	99.17	99.17	99.17	-
	Average	98.88	97.63	98.26	−0.63

**Table 4:** Accuracy analysis across buffer sizes for each model in the class-incremental learning scenario, evaluated for buffer sizes ranging from 100 to 500.

Model	Buffer Size 100	Buffer Size 200	Buffer Size 300	Buffer Size 400	Buffer Size 500
X-DER [6]	98.62	99.41	99.73	99.52	99.67
CNN [11] (+Ours)	98.96	99.26	99.48	99.65	99.54
2-Stream CNN [12] (+Ours)	99.17	99.72	99.41	99.62	99.51
CNN-GRU [13] (+Ours)	98.34	99.38	99.53	99.63	99.44

## Data Availability

The raw data supporting the conclusions of this article will be made available by the authors on request.
